# Supplementary material for: Conditional chemoconnectomics (cCCTomics) as a strategy for efficient and conditional targeting of chemical transmission
Source: eLife. 2024 Apr 30;12:RP91927. doi: 10.7554/eLife.91927 (PMC11060718; doi:10.7554/eLife.91927)
Supplement: Figure 3—source data 3. [file elife-91927-fig3-data3.zip › Figure 3 source data3 part 2.zip!/Figure 3-Source data 3-part2/1053f3-1-CRISPResso_Report_t6djfp6u.zip!/CRISPResso_on_1053f3_3/2a.Nucleotide_percentage_quilt.pdf]

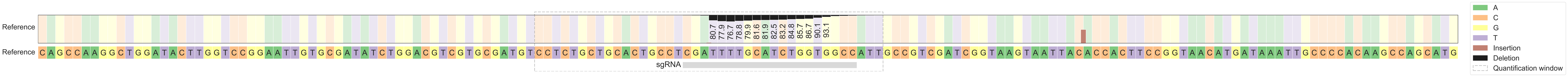

Reference
80.7
77.9
76.7
78.8
79.9
81.6
81.9
82.5
83.2
84.8
85.7
86.7
90.1
93.1
Reference
CAGCCAAGGCTGGATACTTGGTCCGGAATTGTGCGATATCTGGACGTCGTGCGATGTCCTCTGCTGCACTGCCTCGATTTTGCATCTGGTGGCCATTGCCGTCGATCGGTAAGTAATTACACCACTTCCGGTAACATGATAAATTGCCCCACAAGCCAGCATG
sgRNA
A
C
G
T
Insertion
Deletion
Quantification window
